# Isolated Pheochromocytoma in a 73-Year-Old Man With No Clinical Manifestations of Type 1 Neurofibromatosis Carrying an Unsuspected Deletion of the Entire *NF1* Gene

**DOI:** 10.3389/fendo.2019.00546

**Published:** 2019-08-20

**Authors:** Stefanie Parisien-La Salle, Nadine Dumas, Geneviève Rondeau, Mathieu Latour, Isabelle Bourdeau

**Affiliations:** ^1^Division of Endocrinology, Department of Medicine, Research Center, Centre Hospitalier de l'Université de Montréal (CHUM), Montreal, QC, Canada; ^2^Division of Genetics, Department of Medicine, Research Center, Centre Hospitalier de l'Université de Montréal (CHUM), Montreal, QC, Canada; ^3^Department of Pathology and Cellular Biology, Centre Hospitalier de l'Université de Montréal (CHUM), Montreal, QC, Canada

**Keywords:** neurofibromatosis, deletion, phenotype, pheochromocytoma, hypertension

## Abstract

Pheochromocytomas (PHEOs) are a rare cause of endocrine hypertension that requires genetic counseling since at least 30% of PHEOs are associated with a germline mutation in a susceptibility gene. Neurofibromatosis type 1, *NF1* is amongst the 16 known causing genes for pheochromocytomas/paragangliomas. We report a case of a 73-year-old man with PHEO in whom genetic testing revealed a large pathogenic heterozygous deletion of 1.14 Mb encompassing the entire coding sequence of the *NF1* gene while the patient showed no signs of clinical NF1.This case illustrates that the diagnosis of NF1 should not be excluded in patients with PHEO in the absence of clinical diagnosis of the disease and support that older patients with PHEO should also be offered genetic counseling.

## Introduction

Pheochromocytomas (PHEOs) are rare catecholamine-secreting tumors that can cause endocrine hypertension with a prevalence of 0.4–2% in hypertensive patients (1). Germline mutations can be identified in at least 30% of patients with PHEOs and paragangliomas (2). In light of this, genetic testing is now recommended for all patients diagnosed with PHEO (3, 4). Some of these mutations can cause hereditary syndromes such as neurofibromatosis type 1 (NF1), von Hippel-Lindau (VHL), and multiple endocrine neoplasia type 2 (MEN2) (2). NF1 is a neurocutaneous disease caused by a mutation in the *NF1* gene that encodes for the neurofibromin protein (5). The *NF1* gene is located on the chromosome 17q11.2 (2). Two of the following criteria are needed to establish the diagnosis of NF1: inguinal or axillary freckling, two or more neurofibromas, six or more café-au-lait macules, two or more Lisch nodules, optic gliomas, distinctive bone lesions, or a first degree relative with NF1 (6). We report here the case of a man with pheochromocytoma that was diagnosed at 73 years-old carrying an unsuspected *NF1* gene deletion but with no clinical manifestations of NF1.

## Case Report

**Figure 1:**
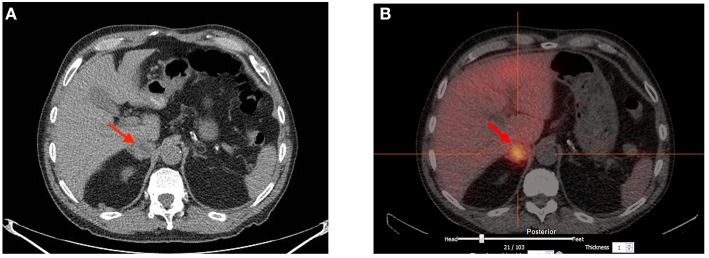
**(A)** Computed Tomography scan showing a 3 cm heterogeneous right adrenal mass of undetermined significance. **(B)** Strong Meta-iodobenzylguanidine (MIBG) uptake of the 2 × 2 × 3 cm right adrenal mass with no evidence of distant metastasis.

**Figure 2:**
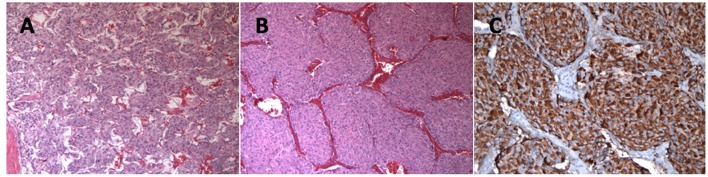
**(A)** More typical Zellballen's architecture with small tumor nests and very focal tumor cell spindling (Hematoxylin and eosin staining, 100 x). **(B)** Large tumor nests were also present (Hematoxylin and eosin staining, 100 x). **(C)** Chromogranine A immunostaining showing strong granular cytoplasmic positivity in the tumor cells (Chromogranine A staining, 200 x).

Following coronary artery bypass grafting, a 73-year-old man presented with symptoms of palpitation, headache, sweating, and blurred vision. His initial systolic blood pressure was over 240 mmHg. The patient's medical history included type 2 diabetes, dyslipidemia, coronary heart disease, prostate cancer and long-standing hypertension that used to be well-controlled with 2 medications; Metoprolol 25 mg twice daily and Valsartan 80 mg daily. However, his hypertension had been harder to control for the past 2 years. His family history was unremarkable except for cases of sudden death for his maternal uncle and aunts. No familial syndromes were noted. Patient was worked up for severe postoperative hypertension with a computed tomography (CT) scan which revealed a small right adrenal mass measured at 31 mm (Figure 1). Biochemical testing included measurements of urinary fractionated metanephrines which revealed high normetanephrines with a value of 880 nmol/d (*N* < 240). However, metanephrines (148 nmol/d: *N* < 275), dopamine (936 nmol/d: N <2570), epinephrines (undetectable: *N* < 110), and norepinephrines (456 nmol/d: *N* < 440) were within the normal limits. Plasma free normetanephrines were 2.10 nmol/L (N <1.20) and free metanephrines 0.4 nmol/L (*N* < 0.48). The patient had normal calcitonin levels. A meta-iodobenzylguanidine (MIBG) scan showed intense and moderate uptake of a 2 × 2 × 3 cm mass of the right adrenal mass with no distant metastases. The patient underwent adrenalectomy and the diagnosis of PHEO was confirmed by histology post-operatively (Figure 2). PHEO of the Adrenal gland Scaled Score (PASS) was 4, and the ki67 proliferation index was less than one percent.

## Methods

After genetic counseling, the patient gave written consent for genetic analysis. Leucocyte DNA was obtained. A genetic panel of 14 susceptibility genes for PHEOs and paragangliomas was performed. The following genes were evaluated for sequence changes and exonic deletions/duplications (*VHL, RET, NF1, SDHD, SDHB, SDHC, SDHA/SDHAF2, TMEM127, MAX, FH, EGLN1, KIF1B, MEN1*) (Invitae, San Francisco, CA). Following the identification of a gross deletion, a comparative genomic hybridization (CGH) was performed using Agilent-CGXTM-HD, 4 × 180K oligonucleotide array (CHU Ste Justine, Unité Biologie Médicale: Génétique, Montréal, Quebec) to identify the boundaries of this genetic deletion. A written informed consent was obtained from the patient for the publication of this case report.

## Results

**Figure 3:**
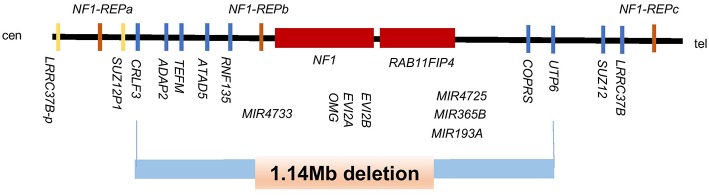
Schematic representation of the genomic region at 17q11.2 harboring the *NF1* gene and its flanking genes. The deletion of 1.14-Mb is shown with the blue bar and includes 11 OMIM genes (C*RLF3, ATAD5, TEFM, ADAP2, RNF135, OMG, EVI2B, EVI2A, NF1, RAB11FIP4, MIRI193A*) and, 7 other genes *(DPRXP4, MIR4733, MIR4724, MIR4725, MIR365B, CORPS, UTP6)*. Modified from Kehrer-Sawatzki et al. (7).

Panel genetic testing revealed a gross heterozygous germline deletion of the genomic region encompassing the full-coding sequence of the *NF1* gene (NM_000267.3). In order to better identify the boundaries and the extends of this event CGH array was performed. The analysis showed a deletion of 15 oligonucleotides at 17q11.2 (position 29, 116, 494-30, 260, 501) (Genome Browser UCSC 2009hg19 assembly). The size of this large pathogenic heterozygous deletion is estimated at 1.14 Mb. In addition to the *NF1* gene, this deletion includes 10 OMIM genes: *CRLF3, ATAD5, TEFM, ADAP2 RNF135, OMG, EVI2A, RAB11FIP4, MIR193A* and 7 other genes: *DPRXP4, MIR4733, MIR4724, MIR4725, MIR365B, COPRS, UTP6* (Figure 3). Patient was re-examined in light of these findings but showed no signs of clinical neurofibromatosis type 1 except for a pectum excavatum that may be associated with NF1.

## Discussion

To our knowledge, we report the oldest patient carrying a large deletion encompassing the entire *NF1* gene in the context of an apparently sporadic pheochromocytoma. Very recently, a case series described three patients with PHEOs carrying unsuspected *NF1* mutations (2). In contrast to our case, these patients were younger (22, 39, and 56 years) and carried only point mutations in the *NF1* gene rather than the loss of the entire gene. In this report, only one of the three patients had discreet signs of NF1 on a repeat examination (2) (Table 1). This shows that a clinical examination is insufficient to exclude a NF1 diagnosis in a case of PHEO.

**Table 1:** Clinical and biochemical descriptions of patients with PHEO and NF1 without clinical NF1 diagnosis described previously and including the current report.

	**Gieldon et al**. **(****2****)**			**Parisien-La Salle et al., 2019 (current article)**
	**Case 1**	**Case 2**	**Case 3**	**Case 4**
**Sex**	**Female**	**Male**	**Female**	**Male**
Past medical history	Hypertension	Prostatic adenocarcinoma, medullary thyroid cancer	None	Hypertension, prostate cancer
Pertinent family history	None	thyroid cancer—type unknown	None	None
Age at NF1 diagnosis (years of age)	53	56	22	73
Age at PHEO diagnosis (years of age)	39 and 53	56	22	73
PHEO location	Bilateral	Bilateral	Left adrenal	Right adrenal
Presenting symptoms	Palpitation, sweating, flushed	Hypertension	Hypertension	Hypertension, palpitation, headache, sweating, and blurred vision
Functionality	Metanephrines and normetanephrines	Metanephrines, normetanephrines, epinephrine, norepinephrines dopamime	Metanephrines, normetanephrines, and methoxytyramine	Normetanephrines
NF1 signs	Macrocephaly	On retrospective examination: Café-au-lait spots, axillary and inguinal freckling, Lisch nodules, neurofibromas and macrocephaly	Short stature	On retrospective examination: pectus excavatum
*NF1* Mutation	*c.6084+1G> A splice-site mutation	*c.7206_7207del (p.His2402Glnfs*4)	*c.7846C> T, p.Arg2616*	Deletion of 15 oligonucleotides 1.14Mb at *17q11.Including the entire coding sequence of *NF1*

* *NM_000267.3*.

In our case, the tumor secreted normetanephrines. NF1 cases of PHEO have been shown to secrete metanephrines or normetanephrines (2). Gross deletions of the *NF1* genes have been described previously in individuals with neurofibromatosis type 1 (8, 9). Large deletions of the *NF1* gene and its flanking regions are frequently associated with a more severe clinical phenotype of NF1 (dysmorphic features, overgrowth, hypotonia, and cognitive impairment) that was not present in this patient (7).

NF1 is a heterogeneous disorder affecting ~1/3,000 births (10). This disease causes multi-organ involvement, which can include PHEOs in 0.1–7% (2, 10). The incidence might be underestimated due to the lack of formal recommendations to universally screen biochemically NF1 patients for PHEOs, as there are in other genetic syndromes such as Von-Hippel-Lindau or MEN2 (10, 11). Recent data showed that 31% of PHEOs in NF1 are found incidentally and only 7.3% were diagnosed following biochemical testing (12). Moreover, PHEOs tend to be diagnosed at an older age in patients with NF1 when compared to other genetic mutations (48 vs. 30 years old) (10).

Diagnosis of NF1 is important as it is associated with an elevated risk of associated malignancies, including malignant peripheral nerve sheath tumors, breast cancer, gastrointestinal stromal tumors, brain, and central nervous system tumors (13). This entails these patients to a more rigorous screening and active surveillance. Furthermore, recognition of NF1 is important for familial screening. In addition to *de novo* mutations NF1 may be inherited (14). The patient reported here did not have a family history of NF1. However, NF1 being an autosomal dominant disorder, appropriate genetic counseling for family members is essential (15). Overall, patients with NF1 need a medical follow-up by a multidisciplinary team to assess and manage complications, counsel on genetic screening (6).

Our findings support the recommendation of more recent guidelines that all patients, independently of age and the clinical presentation of PHEO, should be offered genetic counseling (3, 4). This is illustrated in this 73 year-old man carrying an unsuspected major NF1 deletion. Moreover, genetic testing should not only include evaluation for gene sequence changes but also gene deletion/duplication. Finally, the diagnosis of NF1 should not be excluded in patients with PHEO in the absence of clinical diagnosis of the disease.

## Data Availability

This manuscript contains previously unpublished data. The name of the repository and accession number(s) are not available.

## Author Contributions

SP-L collected the data and wrote the manuscript with the support of IB. ND genetic counselor that met the patient. GR endocrinologist that was involved in patients' care. IB supervised the project and wrote the manuscript with the support of SP-L. ML pathologist that analyzed the specimen and provided the pictures of the tumors' histology. All authors discussed the results and contributed to the final manuscript.

### Conflict of Interest Statement

The authors declare that the research was conducted in the absence of any commercial or financial relationships that could be construed as a potential conflict of interest.

## Abbreviations

PHEO: Pheochromocytoma
NF1: Neurofibromatosis type 1.

## References

[1] Ariton M Juan CS AvRuskin TW. Pheochromocytoma: clinical observations from a Brooklyn tertiary hospital. Endocr Practice. (2000) 6:249–52. 10.4158/EP.6.3.249 11421540

[2] Gieldon L Masjkur JR Richter S Darr R Lahera M Aust D. Next-generation panel sequencing identifies NF1 germline mutations in three patients with pheochromocytoma but no clinical diagnosis of neurofibromatosis type 1. Eur J Endocrinol. (2018) 178:K1–9. 10.1530/EJE-17-0714 29158289

[3] Toledo RA Burnichon N Cascon A Benn DE Bayley JP Welander J. Consensus Statement on next-generation-sequencing-based diagnostic testing of hereditary phaeochromocytomas and paragangliomas. Nat Rev Endocrinol. (2017) 13:233–47. 10.1038/nrendo.2016.185 27857127

[4] Lenders JW Duh QY Eisenhofer G Gimenez-Roqueplo AP Grebe SK Murad MH. Pheochromocytoma and paraganglioma: an endocrine society clinical practice guideline. J Clin Endocrinol Metab. (2014) 99:1915–42. 10.1210/jc.2014-1498 24893135

[5] Cimino PJ Gutmann DH. Neurofibromatosis type 1. Handb Clin Neurol. (2018) 148:799–811. 10.1016/B978-0-444-64076-5.00051-X 29478615

[6] Ferner RE Huson SM Thomas N Moss C Willshaw H Evans DG. Guidelines for the diagnosis and management of individuals with neurofibromatosis 1. J Med Genet. (2007) 44:81–8. 10.1136/jmg.2006.045906 17105749 PMC2598063

[7] Kehrer-Sawatzki H Mautner VF Cooper DN. Emerging genotype-phenotype relationships in patients with large NF1 deletions. Hum Genet. (2017) 136:349–76. 10.1007/s00439-017-1766-y 28213670 PMC5370280

[8] Rasmussen SA Colman SD Ho VT Abernathy CR Arn PH Weiss L. Constitutional and mosaic large NF1 gene deletions in neurofibromatosis type 1. J Med Genet. (1998) 35:468–71. 10.1136/jmg.35.6.468 9643287 PMC1051340

[9] Kayes LM Burke W Riccardi VM Bennett R Ehrlich P Rubenstein A. Deletions spanning the neurofibromatosis 1 gene: identification and phenotype of five patients. Am J Hum Genet. (1994) 54:424–36. 8116612 PMC1918114

[10] Moramarco J El Ghorayeb N Dumas N Nolet S Boulanger L Burnichon N. Pheochromocytomas are diagnosed incidentally and at older age in neurofibromatosis type 1. Clin Endocrinol. (2017) 86:332–9. 10.1111/cen.13265 27787920

[11] Petr EJ Else T. Pheochromocytoma and Paraganglioma in Neurofibromatosis type 1: frequent surgeries and cardiovascular crises indicate the need for screening. Clin Diabetes Endocrinol. (2018) 4:15. 10.1186/s40842-018-0065-4 29977594 PMC6013983

[12] Gruber LM Erickson D Babovic-Vuksanovic D Thompson GB Young WF Jr Bancos I. Pheochromocytoma and paraganglioma in patients with neurofibromatosis type 1. Clin Endocrinol. (2017) 86:141–9. 10.1111/cen.13163 27460956

[13] Stewart DR Korf BR Nathanson KL Stevenson DA Yohay K. Care of adults with neurofibromatosis type 1: a clinical practice resource of the American College of Medical Genetics and Genomics (ACMG). Genet Med. (2018) 20:671–82. 10.1038/gim.2018.28 30006586

[14] Evans DG Howard E Giblin C Clancy T Spencer H Huson SM. Birth incidence and prevalence of tumor-prone syndromes: estimates from a UK family genetic register service. Am J Med Genet Part A. (2010) 152a:327–32. 10.1002/ajmg.a.33139 20082463

[15] Friedman JM Neurofibromatosis 1. In: Adam MP Ardinger HH Pagon RA Wallace SE Bean LJH Stephens K Amemiya A editors. GeneReviews® [Internet]. Seattle, WA: University of Washington (1998).

